# Good Vibrations – Effects of Whole Body Vibration on Attention in Healthy Individuals and Individuals with ADHD

**DOI:** 10.1371/journal.pone.0090747

**Published:** 2014-02-28

**Authors:** Anselm B. M. Fuermaier, Lara Tucha, Janneke Koerts, Marieke J. G. van Heuvelen, Eddy A. van der Zee, Klaus W. Lange, Oliver Tucha

**Affiliations:** 1 Department of Clinical and Developmental Neuropsychology, University of Groningen, Groningen, the Netherlands; 2 Center for Human Movement Sciences, University of Groningen, University Medical Center Groningen, Groningen, the Netherlands; 3 Center of Behaviour and Neuroscience, Department of Molecular Neurobiology, University of Groningen, Groningen, the Netherlands; 4 Department of Experimental Psychology, University of Regensburg, Regensburg, Germany; Hangzhou Normal University, China

## Abstract

**Objectives:**

Most of the current treatment strategies of ADHD are associated with a number of disadvantages which strengthen the need for alternative or additional approaches for the treatment of ADHD. In this respect, *Whole Body Vibration* (WBV) might be interesting as it was found to have beneficial effects on a variety of physiological measures. The present study explored the effects of WBV on attention of healthy individuals and adults diagnosed with ADHD.

**Methods:**

Eighty-three healthy individuals and seventeen adults diagnosed with ADHD participated in the study. WBV treatment was applied passively, while participants were sitting on a chair which was mounted on a vibrating platform. A repeated measure design was employed in order to explore potential effects of WBV treatment on attention within subjects. Attention (i.e. inhibitory control) was measured with a color-word interference paradigm.

**Results:**

A period of two minutes of WBV treatment had significant beneficial effects of small to medium size on attention of both healthy individuals and adults with ADHD. The effect of WBV treatment on attention did not differ significantly between groups.

**Conclusions:**

WBV was demonstrated to improve cognitive performance of healthy individuals as well as of individuals with ADHD. WBV treatment is relatively inexpensive and easy to apply and might therefore be of potential relevance for clinical use. The application of WBV treatment as a cognitive enhancement strategy and as a potential treatment of cognitive impairments is discussed.

## Introduction

Attention deficit hyperactivity disorder (ADHD) is associated with a reduced quality of life [1]. Children and adults with ADHD frequently experience problems in social interaction, which can lead to social rejection, social isolation and discrimination [2]–[5]. Moreover, ADHD is associated with lower academic achievement which was shown to be directly linked to ADHD symptoms and cognitive deficits [6]. With regard to cognition of adults with ADHD, neuropsychological research revealed problems in attention and executive dysfunctions as core features of ADHD, including problems in working memory, inhibitory control, distractibility, vigilance, set-shifting, and task planning [7]–[13]. Accordingly, subjective experiences of inattention and executive dysfunctioning were shown in a considerable proportion of adults with ADHD by using self-report instruments [14].

Conventional behavioral and pharmacological treatment strategies of adults with ADHD include, beside others, cognitive behavioral therapy (CBT) and stimulant drug treatment (e.g. using methylphenidate). Both intervention strategies were shown to have beneficial effects on adults with ADHD, although the first-line choice of treatment strategies is often a stimulant drug therapy [15]–[18]. However, pharmacological treatment of ADHD is associated with several disadvantages for the individuals. For example, it was found that a pharmacological treatment may lead to unsatisfactory effects in a number of patients [17], [19]. In particular, clinical side effects can occur together with stimulant medication, such as insomnia, dry mouth, decreased appetite, and headache [20]. Furthermore, it was demonstrated that methylphenidate improves cognitive functions of adults with ADHD, but does not normalize their level of performance [11], [21]. Consequently, there is a need for alternative or additional treatment options which (1) can be given in addition to the effective treatments (e.g. no interaction with stimulant drug treatment), (2) are time and cost effective, (3) have no detrimental side effects and (4) are effective on symptoms that are most detrimental to the patients’ functioning, such as problems related to attention [13], [22]. To address this need for further treatment options, the present study explores whether *Whole Body Vibration* (WBV) can be a beneficial approach to the treatment of adult ADHD.

WBV can be described as a training method which exposures the whole body of an individual to low frequency environmental vibration. In contrast to the use of WBV in combination with dynamic exercises in the fields of sports and fitness, a passive application of WBV is realized by holding static poses (i.e. standing or sitting) during the time of exposure to vibration. Beneficial effects of WBV were found on various physiological measures, including balance, mobility, posture control, oxygen uptake, heart rate, blood pressure, blood flow and muscle strength [23]–[27]. Moreover, recent animal research provided evidence for a potential value of WBV in improving cognition, as indicated by improved maze learning and an enhanced neuronal activity in mice following the application of WBV treatment [28], [29]. The effect of WBV on cognition in humans, however, was not examined in detail and therefore remains widely unsolved. Several studies applied WBV on healthy individuals and examined whether cognitive functioning was different when participants performed cognitive tasks *while* they received WBV. While some studies did not find any effect of WBV on cognition (i.e. on short-term memory and reasoning skills) [30], [31], others revealed impaired short-term memory, long-term memory and arithmetic reasoning skills while WBV was applied [32]–[34]. A field study to predict the effects of WBV on professional drivers exposed participants to different levels of WBV by testing them in a van which was driving either on asphalt or cobblestone or when the van was halted. Participants had to perform a visual cognitive task and showed a decreased performance while being exposed to WBV compared to a condition of no WBV (van halted) [35]. In contrast to these detrimental effects of WBV on cognition, WBV has also been suggested as an alerting system for improving vigilance [36]. It was argued that particular bands of vibration frequencies cause individuals to increase muscle tension which might facilitate alertness [36]. A study conducted by Mueller and colleagues [37] showed positive effects of passive vibration (proprioceptive stimulation) of the forearm on both neuropsychological performance (as assessed with a computerized attention task) and neurophysiological measures (as assessed with event-related potentials) in patients with traumatic brain injury (TBI) as well as healthy individuals. In this study, proprioceptive stimulation was found to improve task performance of a collapsed group of patients with TBI and healthy individuals. Moreover, patients with TBI showed longer ERP latencies (P300) than healthy individuals. These P300 latencies were shortened by vibratory stimuli in patients with TBI, but not in healthy individuals. This study, therefore, demonstrated that passive vibration may have the potential to improve pathological cognitive processes in patients with neurological conditions and may thus be of relevance for the treatment of cognitive dysfunctions and the field of cognitive rehabilitation. So far, the majority of studies applied WBV at the same time as the cognitive tasks were performed whereas prolonged effects of WBV on subsequent cognitive functioning have not been examined. This is surprising (1) as it appears plausible that vibratory stimuli can cause distraction when they are performed during cognitive testing and (2) when considering that the prolonged effects of WBV are of particular clinical relevance.

Thus, the aim of the present study was to explore the effects of WBV on attention in a group of healthy individuals and in a clinical sample of individuals with cognitive dysfunctions, i.e. adults with ADHD. The potential value of WBV as a cognitive enhancement strategy and its application for the treatment of cognitive dysfunctions are discussed.

## Methods

### Participants

Participation in the study was announced to undergraduate students of the University of Groningen, the Netherlands. The description of the study objectives emphasized that the aim of the study was to examine cognition of typically developed students as well as of students with problems of attention. Therefore, a considerable number of students diagnosed with ADHD indicated their interest to participate in the study. In total, 83 healthy individuals and 17 individuals with ADHD took part in the experiment. All participants were undergraduate students from the University of Groningen, the Netherlands. Participation was voluntary and no monetary compensation was offered.

Healthy individuals (43 female, 40 male) had a mean age of 22.5 years (SD = 3.7 years), ranging from 18 to 31 years. The self-reported body height of individuals ranged from 163 cm to 205 cm (M = 179 cm, SD = 10 cm), with a body weight ranging from 50 kg to 105 kg across individuals (M = 72 kg; SD = 12 kg). Furthermore, the healthy individuals indicated to spend on average 3.8 hours per week on active exercise (SD = 3.6 hours), which ranged from no active exercise at all (0 hours) to 20 hours per week across individuals. None of the healthy individuals reported any history of neurological or psychiatric diseases and none were currently treated with medication known to affect the central nervous system. Furthermore, none of the healthy individuals indicated to suffer from red-green color deficiency which was a prerequisite to perform the Stroop Color-Word Interference task (see below). In addition, all healthy individuals successfully completed two plates of the Ishihara Color Test [38] in order to ensure unimpaired red-green color perception [39].

Individuals with ADHD (9 female, 8 male) had a mean age of 24.2 years (SD = 1.9 years), ranging from 21 to 28 years. According to self-reports, the body height ranged from 159 cm to 190 cm (M = 176 cm, SD = 8 cm) with a body weight ranging from 45 kg to 101 kg (M = 75 kg; SD = 17 kg) across individuals with ADHD. Furthermore, individuals with ADHD reported to spend in average 3.2 hours per week on active exercise (SD = 5.1 hours), ranging from no exercise at all (0 hours) to 20 hours per week across individuals. None of the individuals with ADHD indicated to suffer from red-green color deficiency which was again ensured by successful completion of two plates of the Ishihara Color Test [38], [39]. Moreover, all individuals in the ADHD group reported that they had previously been diagnosed with ADHD by a qualified psychologist or physician. The diagnosis of ADHD was confirmed by a clinical psychiatric interview with an experienced psychologist belonging to the research group. The diagnostic interview involved a retrospective diagnosis of an ADHD in childhood and currently as devised by Barkley and Murphey [40]. In addition, individuals in the ADHD group qualified for inclusion on the basis of four self-report measures for ADHD symptoms. All patients completed the self-report ADHD Rating Scale (ARS, DSM-IV) for retrospective symptoms in childhood (patients fulfilled in average 6.1 criteria for inattention and 6.1 criteria for hyperactivity/impulsivity) and the self-report ADHD Rating Scale (ARS, DSM-IV) for current symptoms (patients fulfilled in average 6.8 criteria for inattention and 5.9 criteria for hyperactivity/impulsivity). Moreover, individuals with ADHD completed the short form of the Wender Utah Rating Scale for childhood ADHD symptoms (WURS) (M = 40.2; SD = 11.0) and the ADHD self-report scale for current symptoms (M = 30.8; SD = 8.6) [41]–[44]. In the diagnostic assessment of the 17 individuals with ADHD, 5 individuals met DSM-IV criteria for ADHD – predominantly inattentive type (ADHD-I), 1 patient met criteria for ADHD – hyperactive-impulsive type (ADHD-H) and 11 individuals met criteria for ADHD – combined type (ADHD-C). Moreover, one of the individuals with ADHD reported to suffer from comorbid disorders (anxiety and depression) and was therefore continuously taking antidepressant medication (including the time of the assessment) as prescribed by the attending physician (150 mg per day). Furthermore, four individuals with ADHD were currently treated with methylphenidate and received individually tailored and clinically appropriate doses with a mean dose of 40.5 mg per day, with individual doses ranging from 10 mg to 72 mg. Pharmacological treatment was not discontinued for this study so that the individuals were on their usual prescribed medication at the time of their participation.

Healthy individuals and individuals with ADHD did not differ significantly in either of the demographic variables, including age (t(98) = 1.778, p = .079), gender (χ^2^ (1) = 0.127; p = .721), body height (t (98) = 1.072, p = .286), body weight (t (98) = 0.825, p = .411) and amount of exercise (t (98) = 0.555, p = .580).

## Materials

### Self-report Measures of ADHD Symptoms

Two scales of the self-report ADHD Rating Scale (ARS, DSM-IV) were applied, one for current symptoms and one for retrospective symptoms in childhood. The ADHD Rating Scale is a self-report instrument for ADHD symptoms made up of the 18 DSM-IV criteria for ADHD [41], [43]. In the Dutch version of the ADHD Rating Scale, five DSM-IV criteria containing double statements were rephrased into two statements, so that the total number of items was 23. In the analysis of the number of fulfilled symptoms, the scores based on the 23 items were recalculated to the original 18 DSM-IV criteria. The items were rated on a 4-point scale (0 = *rarely or never*, 1 = *sometimes*, 2 = *often*, 3 = *very often*) based on participant’s behavior in the past six months (scale for current symptoms) or based on the participant’s behavior in childhood up to twelve years in age (scale for retrospective symptoms). A symptom was considered to be present, if the answer given to the item was *often* or *very often* (score 2 or 3). There were two dimensions, one for inattentive symptoms (nine criteria) and one for hyperactive-impulsive symptoms (nine criteria). To qualify for a clinical diagnosis of adult ADHD, individuals must have met six or more of the nine DSM-IV criteria of inattention and/or hyperactivity-impulsivity in childhood, and at least five of the nine DSM-IV criteria of inattention and/or hyperactivity-impulsivity in adulthood [43].

Furthermore, two self-report scales were applied in order to quantify ADHD symptomatology currently and retrospectively. Childhood ADHD symptoms were self-rated with the short version of the Wender Utah Rating Scale (WURS). The WURS contains 25 items which are rated on a five-point Likert scale [44], [45]. Severity of current ADHD symptoms was self-rated with the ADHD self-report scale [44]. This scale consists of 18 items which correspond to the diagnostic criteria of DSM-IV and which are rated on a four-point Likert scale [44], [46]. A sum score was calculated for each rating scale.

### Whole Body Vibration

**Figure 1:**
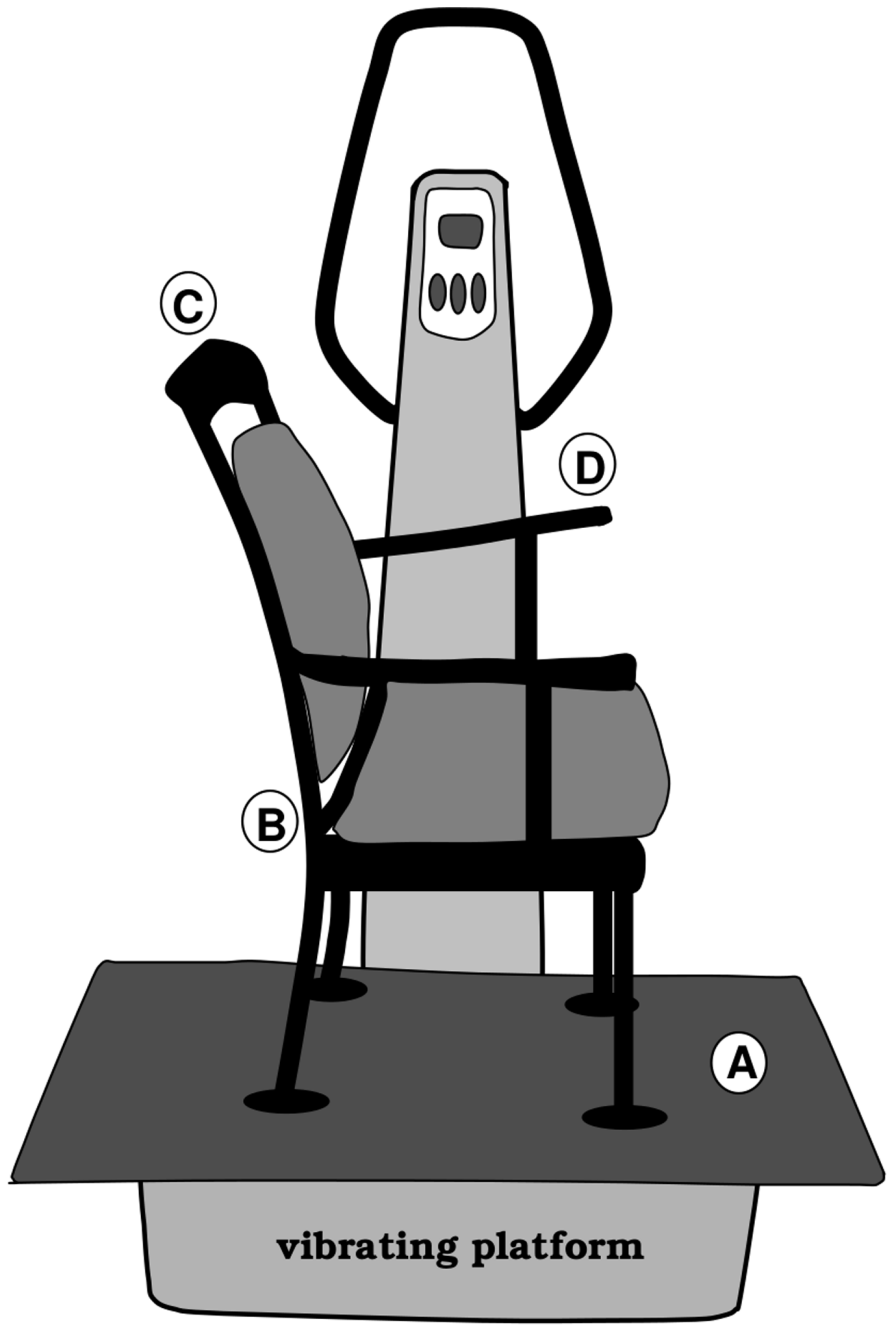
Drawing of the vibrating platform with the mounted wooden platform and chair. Accelerations were measured at location A, B, C and D to determine the actual vibration frequency and amplitude.

Passive WBV was applied by using a vibrating platform (*Vibe 300,* Tonic Vibe, Nantes, France). A wooden platform (0.5 m×0.9 m) was mounted on the *Vibe 300* to enlarge the platform. On top of the platform, a wooden chair was mounted (Figure 1). Both the wooden platform and the chair were attached to the *Vibe 300* with bolts from underneath the *Vibe 300* in order to keep deviations from the vibrating frequency and amplitude on a minimum. The manufacturer settings of *30 Hz vibration frequency* and *4 mm vibration amplitude* were applied as a previous study showed this setup to be comfortable for the participant as well as efficient [47]. However, the construction of a wooden platform and chair mounted on the vibrating device was assumed to cause some deviations from the manufacturer settings with regard to the vibration frequency and amplitude. The actual vertical displacements (frequency and amplitude) were therefore measured on different locations of the chair (see A, B, C and D in Figure 1) based on acceleration data without a person sitting on the chair. The measured displacements (frequency/amplitude) were 30 Hz/0.44 mm (location A), 30 Hz/0.44 mm (location B), 30 Hz/0.66 mm (location C) and 30 Hz/0.50 mm (location D). The modified *Vibe 300* was located in a quiet laboratory at the Department of Clinical and Developmental Neuropsychology of the University of Groningen, the Netherlands.

### Measurement of Attention

To explore the effects of passive WBV on attention, the *Stroop Color-Word Interference task* (Stroop, 1935) was performed. The *Stroop Color-Word Interference task* measure an aspect of attention, i.e. inhibitory control [9]. In the present study, two conditions of the *Stroop Color-Word Interference task* were applied, the *Color Block Test* and the *Color-Word Interference Test*. In the *Color Block Test*, 20 squares each printed in one of four possible colors (yellow, blue, green or red), were presented on a card. The participant’s task was to name the colors of the squares as fast as possible. In the *Color-Word Interference Test*, a list of 52 color names was presented (yellow, blue, green or red). Each word was printed in one of four possible colors (yellow, blue, green or red), however, the color ink of the words was different from the color names (e.g. the word green was printed in blue). The participant was requested to name the ink colors of the words as quickly as possible, thereby ignoring the written color name. In both tests, the time to complete the task was measured. An *interference quotient* was calculated by dividing the time needed for the *Color-Word Interference Test* by the time needed for the *Color Block Test*. The *interference quotient* represented a measure of attention (i.e. inhibitory control), with a larger quotient indicating more problems in inhibitory control. 12 parallel versions (for four practice trials and eight experimental trials) were designed in order to apply the tests in a repeated measure design.

### Procedure

At the beginning of the experiment, all participants completed a questionnaire asking for descriptive information including age, sex, body height, body weight, time spent on average per week on exercise as well as red-green color deficiency. Furthermore, participants were asked for any history of psychiatric or neurological diseases as well as pharmacological treatment. For those students who indicated to be diagnosed with ADHD, a psychiatric interview was conducted followed by the completion of four self-report scales of ADHD symptoms. Subsequently, all participants conducted four practice trials of the *Stroop Color-Word Interference task.* For these practice trials, four parallel versions of the task were used, separated by a resting period of three minutes between each time the task was performed. The four practice trials were applied in order to minimize practice effects during the experimental trials.

**Figure 2:**
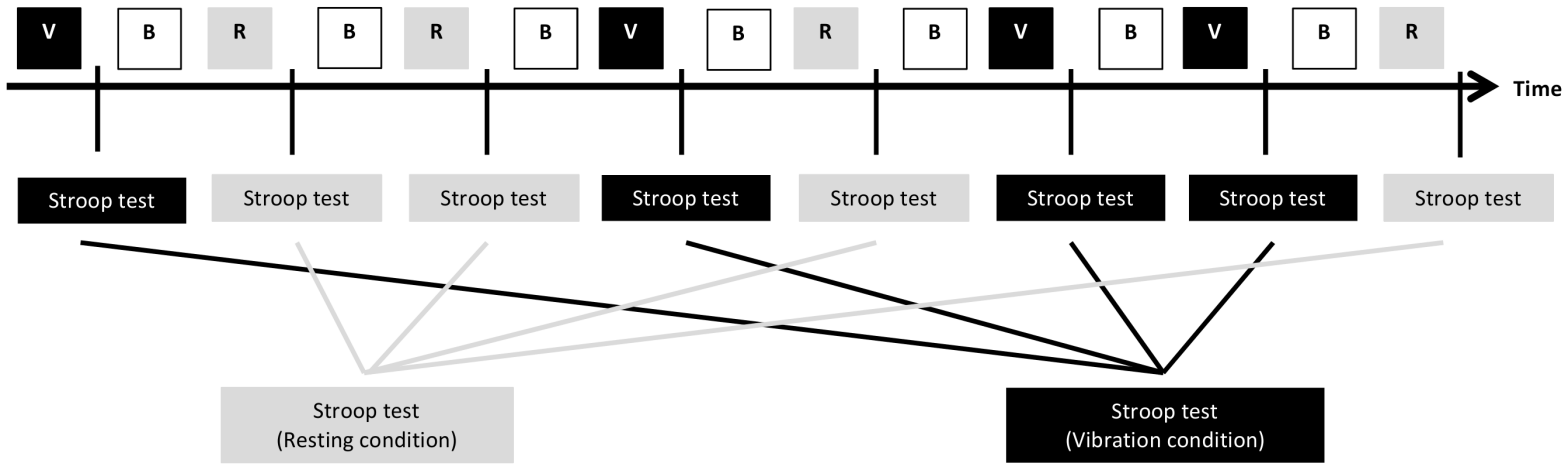
Schematic drawing of the experimental procedure consisting of eight experimental trials. V = Vibration condition: WBV treatment for two minutes; R = Resting condition: No WBV treatment for two minutes; B = Break: Breaks of three minutes (resting); Stroop test = Stroop Color-Word Interference task (Color-Word Interference Test, Color Block Test); For analysis, mean scores were calculated across the four experimental trials for each treatment condition (vibration and resting).

Prior to the first experimental trial, the participant was requested to take seat on the chair mounted on the *Vibe 300*. The participant was instructed to sit in upright position throughout the whole experiment, the arms on the rest and both feet on the wooden platform. The participant was further instructed to keep body movements to a minimum. The experiment consisted of eight experimental trials. Each trial started with a two-minute period of experimental treatment, either a period of vibration (vibration condition) or a resting period of no vibration (resting condition). Immediately after the experimental treatment, the *Color-Word Interference Test* and the *Color Block Test* of the *Stroop Color-Word Interference task* were performed. The *Color-Word Interference Test* always preceded the *Color Block Test.* Subsequently, a resting period of three minutes followed prior to the beginning of the subsequent experimental trial. The eight experimental trials comprised four trials of vibration and four trials of resting. The sequence of the eight experimental trials followed an ABBA-design in order to balance rank order effects (i.e. practice effects) when comparing the two treatment conditions. A schematic drawing of the procedure of eight experimental trials is presented in Figure 2. The duration of the assessment was about 90 minutes.

### Ethics Statement

The study was approved by the Ethical Committee Psychology (ECP) affiliated to the University of Groningen, the Netherlands. All participants signed an informed consent prior to the study and were debriefed after the assessment. All participants were informed that participation was voluntary and that they had the right to refuse or stop participating in the study at any time.

### Statistical Analysis

Since the cognitive task were performed repeatedly (eight times) under two different treatment conditions (vibration or resting), mean scores were calculated across the four trials for each treatment condition, separately for healthy individuals and individuals with ADHD. Nonparametric statistical tests (Wilcoxon signed-rank test for dependent samples) were applied in order to compare cognitive performance (mean interference quotient) between the vibration condition and the resting condition, separately for healthy individuals and individuals with ADHD. A significance level of α = .05 was applied for all comparisons. Moreover, effect sizes (Cohen’s d) were calculated for all comparisons. Following Cohen’s guidelines for interpreting effect sizes (Cohen, 1988), negligible effects (d<0.20), small effects (0.20≤d<0.50), medium effects (0.50≤d<0.80) and large effects (d≥0.80) were distinguished [48].

In addition, the effect of WBV on cognition was compared between healthy individuals and individuals with ADHD. For this reason, ipsative scores were calculated for each individual by subtracting the mean interference quotient of vibration conditions per participant from the mean interference quotient of resting conditions per participant. Ipsative scores, which indicated the effect of WBV on cognition, were then compared between healthy individuals and individuals with ADHD by applying a nonparametric statistical test for independent samples (Mann-Whitney-U-Test).

Furthermore, explorative analyses were carried out in order to examine whether the effects of WBV were determined by demographic variables, including age, gender, body height, body weight and amount of active exercise. This was done by correlating ipsative scores of healthy individuals (indicating the effect of WBV on cognition) with their demographic variables (Spearmen rank correlation). According to Cohen [48], negligible effects (r<0.1), small effects (0.1≤r<0.3), medium effects (0.3≤r<0.5) and large effects (r≥0.5) were distinguished [48]. Finally, gender effects of WBV treatment were analyzed by comparing female and male individuals (Mann-Whitney-U-Test).

## Results

### Effects of WBV Treatment on Attention

**Figure 3:**
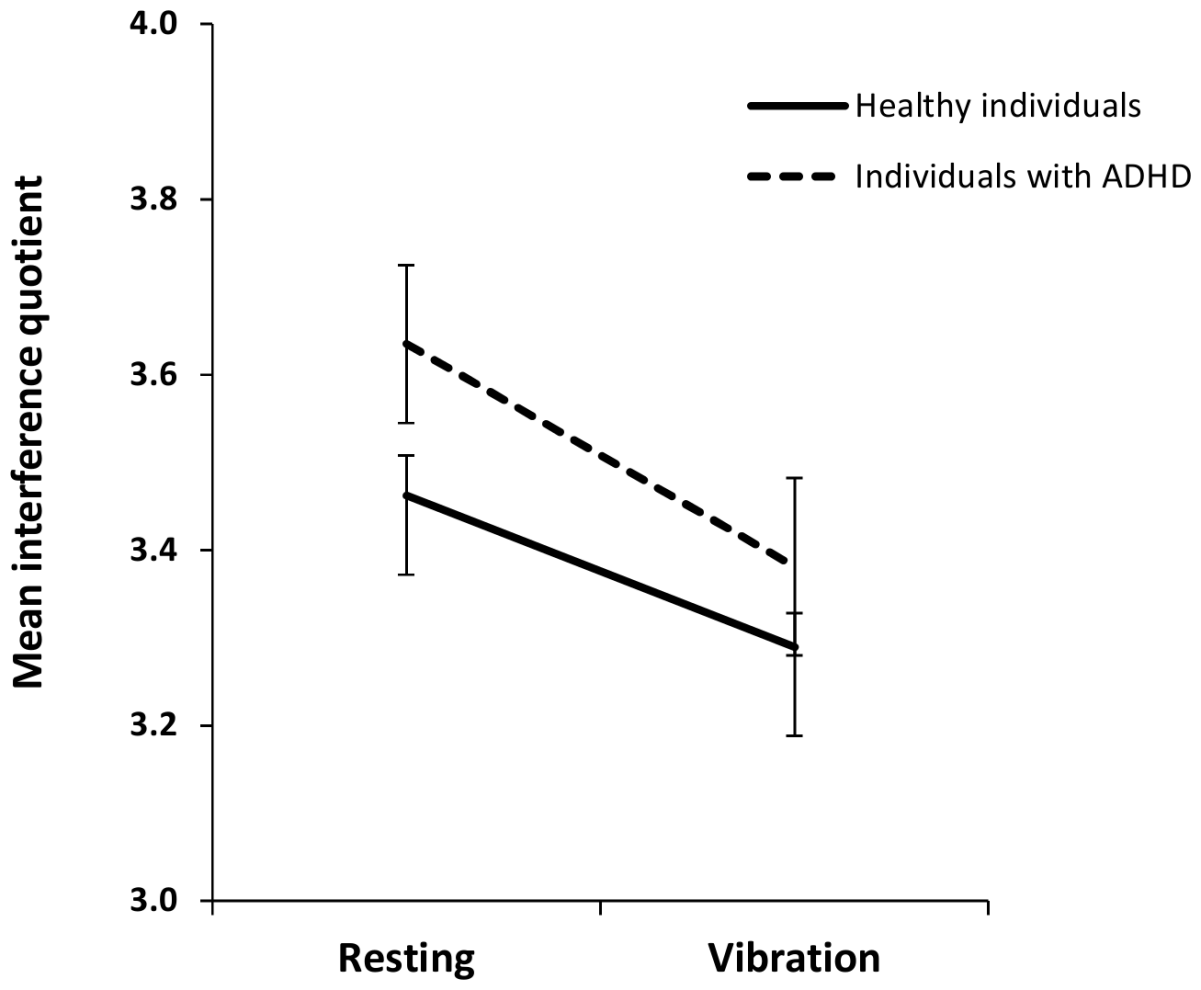
Mean interference quotient for performance of the Stroop Color-Word Interference task following resting and vibration, for both healthy individuals and individuals with ADHD (M±SE).

Data analysis revealed that WBV treatment significantly improved attention performance (M±SD) in both healthy individuals (resting condition: 3.462±0.419; vibration condition: 3.289±0.358; Z = 4.835, p≤.001) and individuals with ADHD (resting condition: 3.635±0.373; vibration condition: 3.381±0.419; Z = 3.243, p = .001). While the difference in healthy individuals was of small size (d = 0.44), a medium effect (d = 0.64) was found in individuals with ADHD (Figure 3).

A separate exploration of the effects of WBV treatment among different subtypes of ADHD showed beneficial effects in all three groups, including patients of the inattentive subtype (n = 5; resting condition: 3.687±0.438; vibration condition: 3.504±0.419), the patient of the hyperactive-impulsive subtype (n = 1; resting condition: 3.055; vibration condition: 2.965) and patients of the combined subtype (n = 11; resting condition: 3.663±0.331; vibration condition: 3.363±0.398).

A comparison of ipsative scores between healthy individuals (n = 83; 0.173±0.293) and the entire group of individuals with ADHD (n = 17; 0.254±0.218) revealed a non-significant difference of small size (Z = 1.542, p = 0.123, d = 0.31).

### Correlations between WBV Treatment Effect and Demographic Variables

Explorative analyses of the association between the effect of WBV treatment and demographic variables in healthy individuals showed that WBV effects were only non-significantly related in small to negligible size to any of the demographic variables including age (r = −.023; p = .836), body height (r = −.178; p = .107), body weight (r = −.091; p = .414) and amount of exercise (r = −.098; p = .380). Female and male individuals did also not differ significantly concerning the effect of WBV treatment as also indicated by a negligible effect size, Z = −1.258; p = .209, d = 0.19.

## Discussion

The present study showed in a large sample of healthy young individuals that WBV treatment has a beneficial effect on subsequent cognitive functioning. Already a short interval of two minutes WBV treatment resulted in an acute beneficial effect on attention (i.e. inhibitory control) as measured with the *Stroop Color-Word Interference task*. Even though evidence of long-term beneficial effects of WBV treatment on cognition is still lacking, the present effects of WBV on attention are promising as the treatment period was rather short (two minutes), emphasizing the significance of the effect. Furthermore, it was assumed that there is only little room for exercise related improvements of cognition in early adulthood [49]. Because the sample of the present study represents a homogenous selection of young, physically healthy and mentally high functioning individuals, WBV treatment appears to have the potential to be an effective cognitive enhancer. This is of high relevance as better cognitive functioning is associated with higher academic achievement and socioeconomic status as well as facilitation of social integration [50] and is further assumed to provide a buffer against cognitive impairment (cognitive reserve) as a consequence of the effects of brain pathology or aging [51]. Demographic changes of the society with an increasing number of older people create considerable concerns (e.g. rise of financial burdens in the health sector) and underline the societal interest in maintaining and improving physical health and cognitive abilities of people. If WBV will be proven to have long-term beneficial effects on cognition, WBV treatment might be a suitable approach to contribute in achieving this goal, in particular, because it is inexpensive, non-invasive, easy to apply and safe (especially when applied passively). For example, studies on the musculoskeletal system demonstrated that WBV can be applied safely on children, older individuals and individuals with neurological and psychiatric conditions including individuals with movement disorders [52]–[57].

In accordance with the studies showing that patients with neurological conditions can benefit from WBV treatment (i.e. in physiological measures, but also cognition) [37], the present study revealed that WBV treatment improves cognitive functioning (i.e. inhibitory control as a measure of attention) of adults diagnosed with ADHD. The effects of WBV treatment on attention in adults with ADHD were of medium size (d = 0.64) and were thus somewhat larger as the effects observed in healthy individuals (d = 0.44). The finding that adults with ADHD benefit more from WBV treatment than healthy individuals cannot be explained by demographic variables since group did not differ in these variables. From a clinical perspective, this finding is very meaningful considering that problems related to attention are common neuropsychological deficits associated with ADHD which has been shown to adversely affect individuals’ occupational functioning and quality of life [1], [11], [58], [59]. Since improved cognitive functions may support social integration and occupational functioning and may also lead to an increased quality of life [1], [11], [58], [59], the present findings consequently stress the potential value of WBV in the treatment of cognitive impairments of individuals with ADHD. An exploration of the effects of WBV treatment among different subtypes of ADHD indicated the largest benefit in patients of the combined subtype, even though small sample sizes hamper a reliable comparison of different subtypes of ADHD and therefore requires replication in larger samples. Furthermore, and more importantly in this context, it has to be emphasized that the effects of WBV treatment on subsequent attention performance as shown in the present study are promising but do not yet justify a recommendation for clinical use. Future studies are required examining long-term effects of WBV treatment on cognition by implementing a variety of control conditions (e.g. vibration of specific body parts only or presentation of auditory stimuli) in order to explore the specific mechanism of WBV treatment which causes cognitive enhancement.

Moreover, it has to be emphasized that the present study revealed positive effects of WBV treatment on performance in subsequent cognitive performance and differed in this respect from the majority of previous research in which participants were exposed to WBV at the same time as they performed cognitive tasks [30]–[35]. These studies reported either no effects or revealed even negative effects of WBV on cognition, which is not surprising considering the distraction that vibratory stimuli may have induced to the simultaneous execution of cognitive tasks. Furthermore, the positive effects of WBV on cognition as shown in the present study on a sample of individuals with ADHD support findings reported by Mueller and colleagues [37], who found positive effects of proprioceptive stimulation (vibration) on cognition and event-related potentials in patients with TBI and thereby suggested that vibratory stimuli may be of potential relevance in a supplementary meaning for the treatment of pathologic cognitive functions.

If cognition enhancing effects of WBV treatment can be replicated in controlled trials, there are several advantages related to a clinical application of WBV treatment emphasizing its value as a novel strategy in the treatment of cognitive dysfunctions of individuals with ADHD. First, WBV does not interfere with other interventions (such as pharmacological treatment) and can thus be applied in addition to conventional interventions. Second, WBV is easy to apply and is (in comparison to other methods) cost effective. A WBV device can be set up in private or institutional settings (e.g. at home, in school or at work) and short periods of WBV treatment of only a few minutes per day might be sufficient to cause clinically relevant effects. In addition, a high compliance rate is expected as this approach requires only little individual effort (e.g. in form of active exercise). Third, no detrimental side effects are currently known by the application of passive WBV. Fourth, and most importantly, WBV was found to have positive acute effects on attention (i.e. inhibitory control), an aspect of cognition which is crucial for everyday life functioning and which has been found to be impaired in both children and adults with ADHD [9], [10], [13], [60], [61].

### Limitations and Future Directions

The present study must be viewed in the context of some limitations. First, with regard to the conclusions aiming at clinical application of WBV treatment, it has to be taken into consideration that the cognition-enhancing effects of WBV treatment on ADHD were observed in a rather small sample (n = 17) of high functioning individuals diagnosed with ADHD. All individuals with ADHD were college students, implying a relatively high level of cognitive functioning. Furthermore, at the time of the assessment, four individuals with ADHD reported to be treated with stimulant medication which compromises the conclusions drawn upon the effects of WBV treatment on cognition. For this reason, the generalization of the observed effects to the population of adults with ADHD and individuals with other neuropsychological disorders might be questioned. However, assuming that there is only little room for improvement in high functioning individuals (ceiling effects), significant effects of small to medium size revealed in two intellectually high functioning groups of people (healthy college students and college students with ADHD) underline the potential of WBV for the treatment of neuropsychological dysfunctions. A replication of the present findings in a large group of healthy individuals and individuals with neuropsychological dysfunctions appears necessary. Future studies addressing effects of WBV treatment in healthy individuals would benefit from the application of screening instruments for psychiatric disorders (e.g. for depression or ADHD) in order to obtain more homogeneous groups and to exclude individuals with high scores on respective symptom scales suggesting a psychiatric diagnosis.

Second, improvements of attention were measured directly after WBV treatment has been applied (acute effects). To establish WBV as a treatment approach, data showing longer lasting effects of cognitive enhancement are required. Therefore, sustained effects of WBV on cognition needs to be explored and should be subject of future research. Longitudinal studies should also take effects of vibratory stimuli on other domains than cognition into consideration, as it was reported that chronic exposure to WBV (e.g. in the occupational setting) is associated with alterations of mood, including fatigue, depression and anxiety [62].

Third, the present study focused on a single aspect of cognition, i.e. inhibitory control. However, it remains unsolved whether WBV has a selective effect on only particular aspects of cognition or whether a broad range of cognitive functions can benefit from WBV treatment. Hence, future studies should examine which functions are most sensitive to WBV.

Finally, and fourth, so far only little is known about the mechanisms underlying the WBV induced cognitive enhancement, and the present study does not contribute to the understanding of these mechanisms. The present study compared the effects of a two-minute period of WBV treatment with a resting condition in which no vibratory stimuli were applied. The implementation of a variety of control conditions would be necessary in order to explore which elements of WBV treatment are responsible for the cognition enhancing effect. For example, no information was obtained (1) whether it might be efficient enough to stimulate particular body parts (e.g. the hands) of individuals to induce cognition enhancing effects, (2) which settings of vibratory stimuli induce most beneficial effects (i.e. frequency and amplitude) and (3) to which extent the produced noise of the vibrating device contributes to the present results.

It was hypothesized that WBV changes muscle length, leading to stimulation of muscle spindles and a reflex response of the muscle [24]. This may enhance muscle activity, resulting in increased oxygen uptake and heart rate. Furthermore, other mechanoreceptors (such as free nerve endings) might be stimulated which might induce sensory stimulation in cortical brain areas. Despite these findings, the underlying mechanism, determining factors and the neurobiological basis of the effects of WBV on cognition in humans remain however widely unresolved and should be addressed in future research. A series of animal studies (on different mouse strains) has been performed [28], [29], [63], [64] revealing that WBV treatment enhanced cognition (e.g. spatial memory) in young and aged mice compared to non-vibrated control mice. Examinations of brains indicated that WBV activates/increases various mechanisms, including (I) the activity of the cholinergic system in the forebrain, (II) the transportation of glucose across the blood-brain barrier, (III) expression of immediate early genes (which make neurons more responsive), (IV) production of proteins necessary for neuronal plasticity and (V) the neurogenesis (i.e. generating new neurons in the adult brain). Furthermore, following WBV, (VI) an increased concentration of tyrosine hydroxylase has been found, an enzyme responsible for catalyzing the synthesis of a precursor of the neurotransmitter dopamine. Dopamine has been shown to affect movement, motivation and cognition [65]–[67] and is also assumed to be involved in the pathophysiology of ADHD [68], [69].

In addition, it can be speculated that the beneficial effects on cognition are not only the result of the vibratory stimuli, but also of the noise which is produced by the vibrating device. Unfortunately, a condition controlling for noise was not implemented in the present study design. A recent model based on theoretical considerations on brain arousal and stochastic resonance as well as on empirical evidence suggested that auditory stimuli (background white noise) can be beneficial to cognitive performance [70], [71]. In these studies, it was argued that whereas auditive noise is detrimental to cognition of healthy individuals, a moderate amount of auditive noise might improve cognitive functions in individuals with hypodopaminergic states, such as individuals with ADHD. With regard to the results of the present study, the beneficial effect of WBV treatment on cognition in adults with ADHD is in line with the prediction of this model. However, as a positive effect of WBV treatment on cognition was also shown in healthy individuals, the model of white noise affecting cognition is not supported and does therefore not appear to be the primary mechanism of the cognitive enhancement found in the present study. However, the present findings in combination with the results of Söderlund and colleagues might indicate that sensory stimulation, independent whether it is auditive noise or mechanic vibration, has the potential to improve cognition in individuals suffering from ADHD.
